# Locating an Underwater Target Using Angle-Only Measurements of Heterogeneous Sonobuoys Sensors with Low Accuracy

**DOI:** 10.3390/s22103914

**Published:** 2022-05-22

**Authors:** Jonghoek Kim

**Affiliations:** Electronic and Electrical Department, Sungkyunkwan University, Suwon 16419, Korea; jonghoek@skku.edu

**Keywords:** underwater target localization, bearing-only measurements, heterogeneous sensors, sonar sensing constraints, sonobuoys, bearing sensor

## Abstract

This paper considers locating an underwater target, where many sonobuoys are positioned to measure the bearing of the target’s sound. A sonobuoy has very low bearing accuracy, such as 10 degrees. In practice, we can use multiple heterogeneous sonobuoys, such that the variance of a sensor noise may be different from that of another sensor. In addition, the maximum sensing range of a sensor may be different from that of another sensor. The true target must exist within the sensing range of a sensor if the sensor detects the bearing of the target. In order to estimate the target position based on bearings-only measurements with low accuracy, this paper introduces a novel target localization approach based on multiple Virtual Measurement Sets (VMS). Here, each VMS is derived considering the bearing measurement noise of each sonar sensor. As far as we know, this paper is novel in locating a target’s 2D position based on heterogeneous sonobuoy sensors with low accuracy, considering the maximum sensing range of a sensor. The superiority (considering both time efficiency and location accuracy) of the proposed localization is verified by comparing it with other state-of-the-art localization methods using computer simulations.

## 1. Introduction

In underwater environments, electromagnetic signal is easily dissipated; thus, sound is mainly used for underwater target localization. As a fundamental function for sonar systems, underwater target localization has gained significant attention [1,2,3,4,5,6]. Target localization by measuring the bearing of target’s sound is an important issue in passive target tracking.

This paper handles localizing a non-cooperative target, such that many sonobuoys are located to measure the bearing of the target’s sound. In other words, we consider the case where multiple sonobuoys are located to measure the bearing of an underwater target’s sound. We consider a Directional Frequency Analysis and Recording (DIFAR) sonobuoy, which consists of an omni-directional hydrophone and two dipole sensors oriented orthogonally [7]. The directional information provided by the dipoles makes it possible to estimate the bearing to an underwater acoustic source [7]. A sonobuoy has very low bearing accuracy, such as 10 degrees [8,9]. The details of a sonobuoy system is addressed in [8,10].

The detection of the target’s sound is associated to the estimation of the target’s bearing using multiple hydrophones. How to estimate the bearing of the target sound using multiple hydrophones is discussed in [7,10,11,12,13]. However, this bearing estimation (target detection phase) is not within the scope of our paper.

Each sonobuoy can measure the bearing (azimuth) of the target sound. However, a sonobuoy cannot measure the elevation angle of the target sound. Thus, the depth of a target cannot be estimated using sonobuoy measurements. This implies that 3D position of the target cannot be estimated using sonobuoy measurements only.

We assume that by processing the target sound, each sonobuoy can measure the bearing of the target sound in real time. The bearing measurements of each sonobuoy are transmitted to a base station, where the target’s 2D location, except for the target’s depth, is calculated in real time. By processing the bearing measurements of every sonobuoy, the base station estimates the underwater target’s 2D position, except for the target’s depth, in real time. This paper handles how to estimate the target’s 2D position using the bearing measurements of multiple sonobuoys.

In practice, we can use multiple heterogeneous bearing sensors. Thus, the variance of a sensor noise may be different from that of another sensor. In addition, the maximum sensing range of a sensor may be different from that of another sensor. For instance, the performance of a cylindrical array sonar may be different from that of another sonar, depending on the geometry of array segments [14].

In the considered bearings-only tracking problem, a sonar sensor measures the bearing of the target, but not the elevation of the target. Bearings-only measurements have been widely used for target localization [15,16,17,18]. Bearings-only measurements can be further utilized for tracking a moving target [19,20,21,22,23]. Multiple turn models, such as the Interacting Multiple Model (IMM), were utilized to track a maneuvering target based on bearings-only measurements [19,24].

Bearing measurements can be utilized for tracking a moving target [17,25], such as a target with a constant velocity [26]. The Range-Parameterized Extended Kalman Filter (RPEKF) [16,27] is a Gaussian-sum filter with multiple EKFs, each initialized at an estimated target range. In this way, we can reduce the initial range estimation error. The target position estimate is then calculated by merging all EKF outputs. The RPEKF assumes that the maximum sensing range of the observer is known in advance. Inspired by the RPEKF, we also assume that the maximum sensing range of a sonobuoy is known in advance. The RPEKF considers tracking a target using measurements of a single bearing sensor. In our paper, we handle the case where multiple bearing sensors are used for locating a target.

Considering bearings-only tracking using a single bearing sensor, Ref. [28] addressed the observability analysis. The authors of [28] showed that sensor maneuver is required to get the observability on the target state. In our paper, we utilize multiple bearing sensors to locate a target. Thus, sensor maneuver is not required to observe the target state.

The authors of [29] presented a formation control of multiple bearing sensors for tracking a target based on bearings-only measurements. The authors of [29] assumed that each bearing sensor can measure the noisy target bearing in real time. Ref. [29] showed that uniform angular distribution centered at the target is the optimal formation configuration. However, in practice, the target position is not known a priori. Thus, the bearing sensors cannot form uniform angular distribution centered at the target. Optimal configuration of bearing sensors is not within the scope of our paper.

Consider the case where multiple bearing sensors are used to localize a target. Least Square (LS) estimation with a closed-form solution, was designed in [30,31,32] for target localization using bearings-only measurements. The bearings-only LS suffers from severe bias problems. In [33], an improved LS with bias compensation strategy was developed for bearings-only passive target tracking. Moreover, Ref. [33] assumed that the bearing noise is sufficiently small, such that the first order Taylor series on the bearing noise can be applied. Our paper considers the case where bearing accuracy of sonobuoy sensor is very low; thus, the first order Taylor series on the bearing noise cannot be applied.

The LS was extended to the Weighted Least Square (WLS) estimation in [34]. In the WLS, measurement noise variance was used to enhance the estimation result, assuming that the bearing measurement noises are sufficiently small. The authors of [31,35] extended the LS to the Total Least Square (TLS) estimation algorithm. The TLS was developed based on orthogonal vectors with the advantage of simplicity and reduced bias in the presence of bearing noise and observer position errors. Refs. [31,35] argued that the TLS has better performance than the LS. However, LS [30,31,32], WLS [34], and TLS [31,35] did not consider the maximum sensing range of a sonar sensor. Using MATLAB simulations, we verify the outperformance (considering both location accuracy and computational load) of the proposed localization method by comparing it with LS, WLS, and TLS localization methods.

Several papers [36,37] addressed bearings-only localization using multiple passive sensors, which requires an initial guess for target position. Reference [36] introduced the Gauss Newton (GN) algorithm applied to bearings-only target estimation. The GN method is an iterative method to derive the target estimation. The convergence of the GN estimator is sensitive to the initial guess and the step size [38]. Assuming that bearing measurements and inter sensor bearing measurements are available, Ref. [37] addressed a subspace algorithm which requires an initial guess for target position. In our paper, we do not compare the proposed localization approach with [36,37], since [36,37] require an initial guess for target location. Note that the localization approach proposed in our paper can be used as an initial guess required in [36,37].

We consider a scenario where only a few sonobuoy sensors exist and each sensor has very low bearing accuracy, such as 10 degrees [8]. In order to cope with poor accuracy of sonobuoy sensors, this paper introduces a novel target localization approach based on multiple Virtual Measurement Sets (VMS). Here, each VMS is derived considering the bearing measurement noise of each sonar sensor. Among all feasible target estimates derived from each VMS, we select an estimate which has the minimum residual (the difference between the actual and predicted measurements), such that the estimate satisfies the sonar sensing range constraints. The selected estimation is then utilized as the final target estimation.

Inspired by the RPEKF, we assume that the maximum sensing range (e.g., 15 km which appeared in [9]) of a sonobuoy is known in advance. As far as we know, our paper is novel in localization of a target using heterogeneous sonobuoy sensors with low accuracy, considering the sensing range constraints of a sonar sensor. Our paper is distinct from LS [30,31,32], WLS [34], and TLS [31,35], since our location approach considers the maximum sensing range of a sonar sensor. Using MATLAB simulations, we demonstrate the superiority (considering both time efficiency and location accuracy) of the proposed localization, by comparing it with LS, WLS, and TLS localization methods.

This paper is organized as follows. Section 2 presents the bearings-only target localization. Using MATLAB simulations in Section 3, we verify the performance of the proposed estimation method based on VMS. Section 4 provides the conclusions.

## 2. Bearings-Only Target Localization

Suppose that there are *K* heterogeneous sonobuoy sensors which measure the bearing of the target. We assume that by processing the target’s sound, each sonobuoy can measure the bearing of the target’s sound in real time. How to calculate the target’s bearing using a sonobuoy is discussed in [7,10,11,12,13].

The bearing measurements of each sonobuoy are transmitted to a base station, where the target’s 2D location is calculated in real time. By processing the bearing measurements of every sonobuoy, the base station estimates the underwater target’s 2D position in real time. This paper handles how to estimate the target’s 2D position using the bearing measurements of multiple sonobuoys.

Let $E={\left(x^{t},y^{t}\right)}^{T}$ denote the true target position. Let $\hat{E}$ denote the estimated target position. Let ${\left(x_{k},y_{k}\right)}^{T}$ denote the *k*-th sonobuoy sensor position (k∈{1,2,…,K}). We assume that each sonobuoy sensor is localized using Global Positioning Systems (GPS). Let $\phi_{k}$ present the bearing angle of the target with respect to the *k*-th sonar sensor.

(1) $$\begin{array}{c} \phi_{k}=\phi^{true}+n_{k}. \end{array}$$

Here, $\phi^{true}=atan2\left(y^{t}-y_{k},x^{t}-x_{k}\right)$ represents the true bearing associated to true relative position. In (1), atan2(y,x) is used to represent the phase (angle) of a complex number x+iy. $n_{k}$ in (1) has Gaussian distribution with zero mean and variance $\sigma_{k}^{2}$. Here, $\sigma_{k}\neq \sigma_{k^{\prime }}$ ($k\neq k^{\prime }$) is feasible, since we handle heterogeneous sensors. We assume that $\sigma_{k}$ is known a-priori using experiments with the *k*-th sensor.

The empirical rule states that 99.7 percents of data observed following a normal distribution exists within 3 standard deviations of the mean. Thus, $n_{k}$ in (1) is within the following interval:

(2) $$\begin{array}{c} I\left(\sigma_{k}\right)=\left[-3\times \sigma_{k},3\times \sigma_{k}\right] \end{array}$$

with 99.7 percents probability. In (1), $∥n_{k}∥$ can be considered as Unknown-But-Upperbounded (UBU) by $3\times \sigma_{k}$.

Many papers on bearing-only localization [33,39,40] assumed that the bearing noise is sufficiently small, such that the first order Taylor series on the bearing noise can be applied. This implies that [33,39,40] used

(3) $$\begin{array}{c} \cos\left(\phi_{k}\right)=\cos\left(\phi^{true}+n_{k}\right)\approx \cos\left(\phi^{true}\right)-n_{k}\times \sin\left(\phi^{true}\right),\\ \sin\left(\phi_{k}\right)=\sin\left(\phi^{true}+n_{k}\right)\approx \sin\left(\phi^{true}\right)+n_{k}\times \cos\left(\phi^{true}\right), \end{array}$$

where $\phi^{true}=atan2\left(y^{t}-y_{k},x^{t}-x_{k}\right)$ in (1). Our article considers the case where bearing accuracy is very low; thus, the first order Taylor series on the bearing noise cannot be applied. Therefore, ${\left(n_{k}\right)}^{2}$ cannot be ignored, as in (3).

Let $r_{k}$ denote the maximum sensing range of the *k*-th sensor. Here, $r_{k}\neq r_{k^{\prime }}$ ($k\neq k^{\prime }$) is feasible, since we consider heterogeneous sensors. Inspired by the RPEKF [16,27], we assume that $r_{k}$ is known in advance. Distinct from the RPEKF, we consider the case where there are more than one sensor.

In MATLAB simulations, the maximum sensing range $r_{k}$ is set as 15 km for all k∈{1,2,…,K}. This sensing range information appeared in [9].

### 2.1. Least Square (LS), Weighted Least Square (WLS), and Total Least Square (TLS) Methods

Ignoring the noise term in (1), we have

(4) $$\begin{array}{c} \cos\left(\phi_{k}\right)y^{t}-\cos\left(\phi_{k}\right)y_{k}=\sin\left(\phi_{k}\right)x^{t}-\sin\left(\phi_{k}\right)x_{k}. \end{array}$$

(4) leads to

(5) $$\begin{array}{c} V=B\times E, \end{array}$$

where

(6) $$\begin{array}{c} V=\left(\begin{array}{c} -y_{0}\times \cos\left(\phi_{0}\right)+x_{0}\times \sin\left(\phi_{0}\right)\\ -y_{1}\times \cos\left(\phi_{1}\right)+x_{1}\times \sin\left(\phi_{1}\right)\\ \vdots \\ -y_{K}\times \cos\left(\phi_{K}\right)+x_{K}\times \sin\left(\phi_{K}\right) \end{array}\right),and \end{array}$$

(7) $$\begin{array}{c} B=\left(\begin{array}{ccc} \sin(\phi_{0}), & & -\cos(\phi_{0})\\ \sin(\phi_{1}), & & -\cos(\phi_{1})\\ \vdots & & \vdots \\ \sin(\phi_{K}), & & -\cos(\phi_{K}) \end{array}\right). \end{array}$$

We calculate the Least Square (LS) solution [31,32] as

(8) $$\begin{array}{c} {\hat{E}}_{LS}={\left(B^{T}B\right)}^{-1}B^{T}V. \end{array}$$

The LS was extended to the Weighted Least Square (WLS) estimation in [34]. In the WLS, measurement noise variance was used to enhance the estimation result, assuming that the bearing measurement noises are sufficiently small. We calculate the WLS solution [34] as

(9) $$\begin{array}{c} {\hat{E}}_{WLS}={\left(B^{T}WB\right)}^{-1}B^{T}WV \end{array}$$

where the weight matrix is

(10) $$\begin{array}{c} W=B_{1}\sum B_{1}^{T}. \end{array}$$

Here, ∑ is a diagonal matrix whose diagonal terms are given by $\left[\sigma_{1}^{2},\sigma_{2}^{2},\ldots ,\sigma_{K}^{2}\right]$ in this order. In addition, $B_{1}$ is a diagonal matrix whose diagonal terms are given by $\left[d_{1},d_{2},\ldots ,d_{K}\right]$ in this order. Here, $d_{k}$ is

(11) $$\begin{array}{c} d_{k}=\sqrt{{\left(x^{t}-x_{k}\right)}^{2}+{\left(y^{t}-y_{k}\right)}^{2}}. \end{array}$$

Since $d_{k}$ is not available in practice, we use ${\hat{E}}_{LS}$ for estimation of $\left[x^{t},y^{t}\right]$.

The TLS in [31,35] is an extension of (8). The TLS is given as

(12) $$\begin{array}{c} {\hat{E}}_{TLS}={\left(B^{T}B-\sigma_{s}^{2}I\right)}^{-1}B^{T}V. \end{array}$$

Here, $\sigma_{s}$ is the smallest singular value of matrix [B,V]. Refs. [31,35] showed that the TLS has better performance than the LS. From a numerical analyst’s point of view, (12) indicates that the TLS solution is more ill-conditioned than the LS solution, since it has a higher condition number [31]. The implication is that errors in the data are more likely to affect the TLS solution than the LS solution. This is particularly true for the worst case perturbations [31]. However, from a statistician’s point of view, (12) implies that the TLS method asymptotically removes the bias by subtracting the error covariance matrix (estimated by $\sigma_{s}^{2}I$) from the data covariance matrix $B^{T}B$ [31].

### 2.2. Bearings-Only Target Localization by Simulating Multiple VMSs

#### VMS-Based Localization Method

In order to improve the location accuracy compared to the LS-based solutions (Section 2.1), we develop a novel localization approach of using Virtual Measurement Sets (VMS). Suppose that we use *Q* VMSs in total. Each VMS has *K* virtual measurements in total. The *q*-th VMS (q∈{1,…,Q}) has *K* virtual measurements, say $\left(\phi_{1}^{q},\phi_{2}^{q},\ldots ,\phi_{K}^{q}\right)$. Here, the *k*-th virtual measurement in the *q*-th VMS is generated by adding a random value in the interval $I(\sigma_{k})$ (2) to the bearing measurement of the *k*-th sensor (∀k≤K). Recall we assume that $\sigma_{k}$ is known a-priori using experiments with the *k*-th sensor (k∈{1,2,…,K}).

In the *q*-th VMS, $\phi_{k}^{q}$ (*k*-th virtual measurement) is used to derive the true bearing $\phi^{true}=atan2\left(y^{t}-y_{k},x^{t}-x_{k}\right)$, which is $\phi_{k}-n_{k}$ in (1). By processing the virtual measurements associated to the *q*-th VMS (q∈{1,…,Q}), we generate a feasible target estimate, say ${\hat{Z}}^{q}$.

Among all feasible target estimates ${\hat{Z}}^{q}$ (q∈{1,…,Q}), we select an estimate which has the minimum residual (the difference between the actual and predicted measurements), such that the estimate satisfies the sonar sensing constraints. The selected estimation, say ${\hat{Z}}^{q^{*}}$, is used as the final target estimation.

We present the VMS approach in detail. Each VMS has *K* virtual measurements in total. Let

(13) $$\begin{array}{c} \left(\phi_{1}^{q},\phi_{2}^{q},\ldots ,\phi_{K}^{q}\right) \end{array}$$

denote the virtual measurements of the *q*-th VMS (q∈{1,…,Q}). Using (1), we generate the virtual measurements of the *q*-th VMS (q∈{1,…,Q}) as follows. In the case where *q* is one, we use

(14) $$\begin{array}{c} \phi_{k}^{1}=\phi_{k} \end{array}$$

for all k∈{1,2,…,K}.

Note that the 1-st VMS uses $\phi_{k}$, true bearing measurement, for target estimation. In the case where *q* is not one (i.e., q∈{2,3,…,Q}), we use

(15) $$\begin{array}{c} \phi_{k}^{q}=\phi_{k}+g_{k}^{q} \end{array}$$

for all k∈{1,2,…,K}. Here, $g_{k}^{q}$ is a virtual noise, generated as a random value in the interval $I(\sigma_{k})$ in (2). (15) implies that $\phi_{k}^{q}$ is generated by adding a random value in the interval $I(\sigma_{k})$ to the bearing measurement of the *k*-th sensor (∀k∈{1,2,…,K}).

Using (1), (15) leads to

(16) $$\begin{array}{c} \phi_{k}^{q}=\phi^{true}+n_{k}+g_{k}^{q}. \end{array}$$

Here, $\phi^{true}=atan2\left(y^{t}-y_{k},x^{t}-x_{k}\right)$ is the true bearing associated to true relative position.

As *Q* increases, it is more probable that we have a VMS $\phi_{k}^{q}$ (q∈{2,3,…,Q}) satisfying

(17) $$\begin{array}{c} ∥n_{k}+g_{k}^{q}∥\leq ∥n_{k}∥ \end{array}$$

for all k∈{1,2,…,K}. (17) implies that by adding a virtual noise $g_{k}^{q}$, we can have a VMS $\phi_{k}^{q}$, which is disturbed with smaller noise compared to true noise $n_{k}$.

By applying the LS solution (8) to the virtual measurements (13) of the *q*-th VMS (q∈{1,…,Q}), we generate a feasible target estimate, say ${\hat{Z}}^{q}$. Using (14), ${\hat{Z}}^{1}$ is the LS solution (8) associated to true bearing measurements.

Among all feasible target estimates ${\hat{Z}}^{q}$ (q∈{1,…,Q}), we select an estimate which has the minimum residual, such that the estimate satisfies the sonar sensing constraints. The selected estimation, say ${\hat{Z}}^{q^{*}}$, is used as the final target estimation.

Let ${\hat{Z}}^{q}\left[n\right]$ denote the *n*-th element in ${\hat{Z}}^{q}$. The bearing angle associate to ${\hat{Z}}^{q}$ is

(18) $$\begin{array}{c} {\hat{\phi }}_{k}^{q}=atan2\left({\hat{Z}}^{q}\left[2\right]-y_{k},{\hat{Z}}^{q}1-x_{k}\right). \end{array}$$

Recall that $\phi_{k}$ is the true bearing measurement of the *k*-th sensor. We define the angle deviation $\epsilon_{k}$ as

(19) $$\begin{array}{c} \epsilon_{k}=atan2\left(\sin\left({\hat{\phi }}_{k}^{q}-\phi_{k}\right),\cos\left({\hat{\phi }}_{k}^{q}-\phi_{k}\right)\right). \end{array}$$

Here, atan2(α,β) returns the phase angle of a complex number α+jβ. $\epsilon_{k}$ exists between −π and π. In the case where ${\hat{\phi }}_{k}^{q}-\phi_{k}$ exists between −π and π, $\epsilon_{k}$ is identical to ${\hat{\phi }}_{k}^{q}-\phi_{k}$. Using (19), $\epsilon_{k}=0$ when ${\hat{\phi }}_{k}^{q}=\phi_{k}$.

Using $\epsilon_{k}$ in (19), the residual associated to the *q*-th (q∈{1,2,…,Q}) VMS is set as

(20) $$\begin{array}{c} Res^{q}=\sum_{k=1}^{K}\left(\frac{\epsilon_{k}^{2}}{\sigma_{k}^{2}}+\eta_{k}\right). \end{array}$$

Here, $\sigma_{k}$ is used for normalizing the angle deviation term $\epsilon_{k}$. In addition, $\eta_{k}$ is set as a constant P>0 in the case where

(21) $$\begin{array}{c} ∥{\hat{Z}}^{q}-\left(x_{k},y_{k}\right)∥>r_{k}, \end{array}$$

or the straight line segment connecting ${\hat{Z}}^{q}$ and $\left(x_{k},y_{k}\right)$ crosses an obstacle. Here, we use the fact that underwater sound cannot penetrate an obstacle, such as coastline. This fact can be considered as the obstacle constraint.

In (20), we set $\eta_{k}=0$ if

(22) $$\begin{array}{c} ∥{\hat{Z}}^{q}-\left(x_{k},y_{k}\right)∥\leq r_{k} \end{array}$$

and the straight line segment connecting ${\hat{Z}}^{q}$ and $\left(x_{k},y_{k}\right)$ does not cross an obstacle. $\eta_{k}=0$ implies that ${\hat{Z}}^{q}$ satisfies the range constraint as well as the obstacle constraint.

In (20), P>0 is a tuning parameter, presenting the penalty for sonar sensing constraints. In the case where ${\hat{Z}}^{q}$ does not satisfy the constraints, $\eta_{k}$ in (20) is set as P>0.

We define $q^{*}$ as

(23) $$\begin{array}{c} q^{*}=argmin_{q}Res^{q}. \end{array}$$

Since ${\hat{Z}}^{q^{*}}$ provides the minimum residual among all $Res^{q}$ (q∈{1,2,…,Q}), ${\hat{Z}}^{q^{*}}$ is utilized as the target estimate.

However, there is an exception case. If ${\hat{Z}}^{q^{*}}$ does not satisfy

(24) $$\begin{array}{c} ∥\left(\left(x_{k},y_{k}\right)-{\hat{Z}}^{q^{*}}\right)∥\leq r_{k} \end{array}$$

for any k∈{1,2,…,K}, then ${\hat{Z}}^{q^{*}}$ is not a viable solution. Thus, ${\hat{Z}}^{1}$ is utilized as the final target estimate. Recall that ${\hat{Z}}^{1}$ is the LS-based solution associated to true bearing measurements. If ${\hat{Z}}^{q^{*}}$ satisfies (24) for every k∈{1,2,…,K}, then ${\hat{Z}}^{q^{*}}$ is utilized as the final target estimate.

We next analyze the computational complexity of the VMS method. Since one derives ${\hat{Z}}^{q}$ for all q∈{1,2,…,Q}, the VMS method has complexity O(Q).

## 3. MATLAB Simulations

Using MATLAB simulations, we demonstrate the superiority of the VMS-based localization method in Section 2.2. We consider the marine scenario where multiple sonobuoys are located to measure the bearing of an underwater target’s sound. All sonobuoys are deployed to measure the bearing of the target sound in real time. By processing the bearing measurements of each sonobuoy, our goal is to estimate the target’s 2D position.

We compare among the following methods:

1. TLS method (12).
2. LS method (8).
3. WLS method (9).
4. VMS-LS method (VMS using LS solution (8)).

For generating a virtual noise in (15), we use 3=3. We use the penalty constant as $P=10^{3}$ in (20). The maximum sensing range $r_{k}$ is 15 km for all k∈{1,2,…,K}. This sensing range information appeared in [9].

The performance of a cylindrical array sonar may be different from that of another sonar, depending on the geometry of array segments [14]. Recall that ${\left(x_{k},y_{k}\right)}^{T}$ denotes the *k*-th sonar sensor position (k∈{1,2,…,K}). We consider heterogeneous sonobuoy sensors as follows. In the case where *k* mod 2 is zero, the bearing noise is set as $\sigma_{k}=5$ degrees. In the case where *k* mod 2 is not zero, the bearing noise is set as $\sigma_{k}=10$ degrees. This noise statistic is typical for sonobuoy systems [8].

We use 1000 Monte Carlo (MC) simulations of the scenario. The error in the target estimation at the *i*-th MC simulation is

(25) $$\begin{array}{c} err_{i}=∥{\hat{Z}}_{i}-E∥. \end{array}$$

Here, ${\hat{Z}}_{i}$ is the target estimation at the *i*-th MC simulation. In addition, E is the true position of the target. Since E is not accessible by sonar sensors, $err_{i}$ is not accessible in practice.

Let M(err) (in meters) denote the mean of $err_{i}$ for all i∈{1,2,…,1000}. In other words, we use

(26) $$\begin{array}{c} M\left(err\right)=\frac{1}{1000}\sum_{i=1}^{1000}err_{i}. \end{array}$$

Let RunTime (in seconds) represent the computation time of running MATLAB for each method in one MC simulation. Large RunTime implies that the computational burden of the associated method is large.

### 3.1. Scenario 1

**Figure 1:**
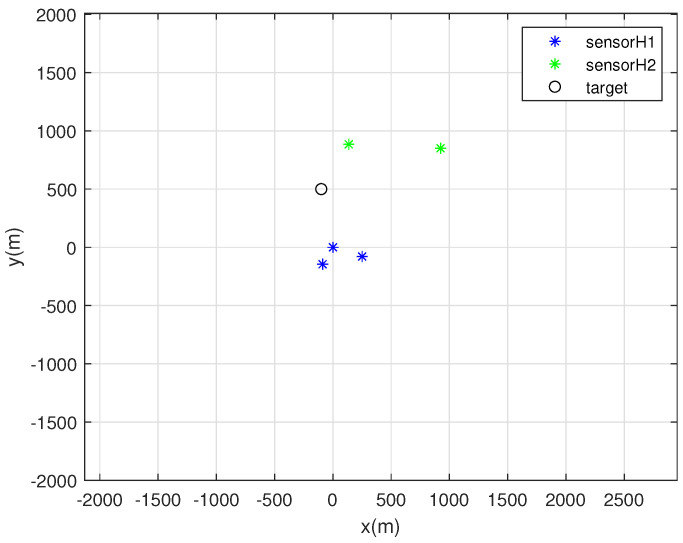
One MC simulation of Scenario 1. At each MC simulation, we randomly deploy 5 sonobuoy sensors inside the circle with radius 2000 m, whose center is at the origin. Each sensor with bearing noise $\sigma_{k}=5$ degrees is marked with a blue asterisk. Every sensor with bearing noise $\sigma_{k}=10$ degrees is marked with a green asterisk. In addition, the true target at (−100, 500) is depicted as a black circle.

One MC simulation of Scenario 1 is depicted in Figure 1. At each MC simulation, we randomly deploy 5 sonobuoy sensors inside the circle with radius 2000 m, whose center is at the origin. This random deployment simulates the case where sonobuoys are deployed by airplanes.

Recall that ${\left(x_{k},y_{k}\right)}^{T}$ denotes the *k*-th sonar sensor position (k∈{1,2,…,K}). For random sensor deployment, we use the following equation.

(27) $$\begin{array}{c} x_{k}=R\times \cos\left(A\right)\\ y_{k}=R\times \sin\left(A\right) \end{array}$$

Here, *R* is a random number selected in the interval [0,2000]. In addition, *A* is a random number selected in the interval [0,2×π].

In Figure 1, each sensor with bearing noise $\sigma_{k}=5$ degrees is marked with a blue asterisk. Furthermore, each sensor with bearing noise $\sigma_{k}=10$ degrees is marked with a green asterisk. In addition, the true target located at (−100, 500) is depicted as a black circle. See that sensors are deployed close to the target, since sensors are deployed inside the circle with radius 2000 m, whose center is at the origin.

**Figure 2:**
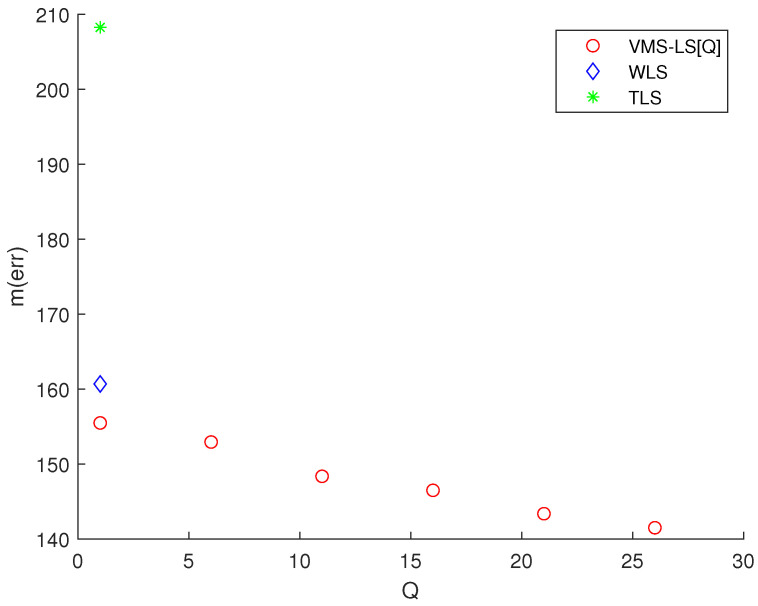
Scenario 1. The variation of M(err) with respect to *Q*. TLS and WLS are plotted at Q=1. The algorithm with the lowest M(err) is VMS−LS[26]. This demonstrates the effectiveness of utilizing VMS−LS proposed in our article.

Considering Scenario 1, Figure 2 presents the simulation results of various algorithms, mentioned at the beginning of Section 3. This figure shows the variation of M(err) with respect to *Q*. TLS and WLS are plotted at Q=1. In Figure 2, VMS−LS[Q] implies that *Q* VMSs are used in the VMS−LS method. Note that using (14), LS solution in (8) is identical to VMS−LS[1].

**Figure 3:**
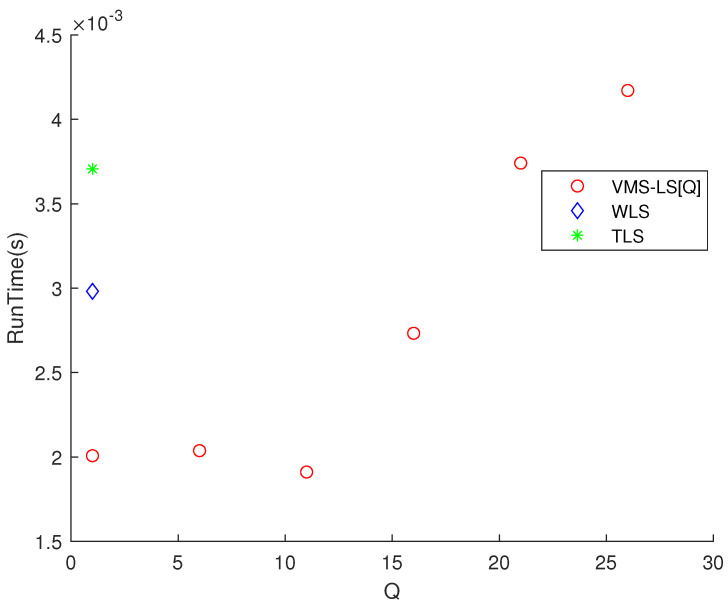
Scenario 1. The variation of RunTime with respect to *Q*. As *Q* increases, computational burden increases in general.

Figure 2 shows that increasing *Q* improves the estimation accuracy of VMS−LS algorithm. The algorithm with the lowest M(err) is VMS−LS[26]. This verifies the performance of using VMS−LS proposed in our paper. Figure 3 shows that as *Q* increases, computational burden increases in general.

### 3.2. Scenario 2

**Figure 4:**
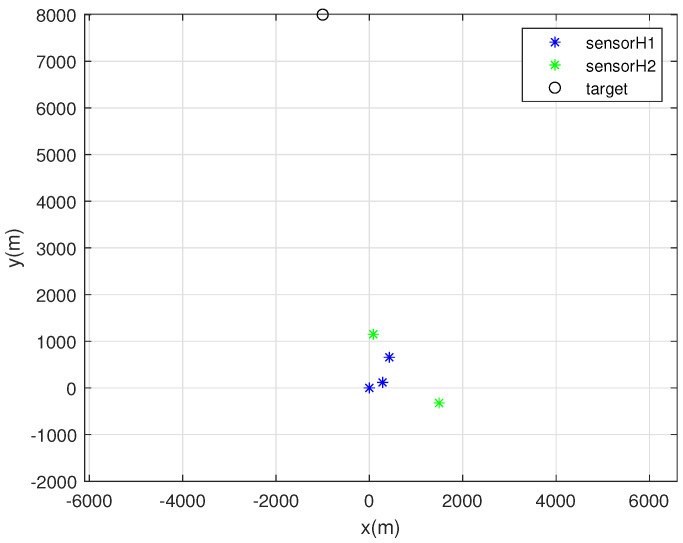
One MC simulation of Scenario 2. At each MC simulation, we randomly deploy 5 sonobuoy sensors inside the circle with radius 2000 m, whose center is at the origin. Each sensor with bearing noise $\sigma_{k}=5$ degrees is marked with a blue asterisk. Moreover, each sensor with bearing noise $\sigma_{k}=10$ degrees is marked with a green asterisk. In addition, the true target position is depicted as a black circle.

One MC simulation of Scenario 2 is depicted in Figure 4. In this scenario, the target exists far from the sensors. At each MC simulation, we randomly deploy 5 sonobuoy sensors inside the circle with radius 2000 m, whose center is at the origin. For random sensor deployment, we use (27). Each sensor with bearing noise $\sigma_{k}=5$ degrees is marked with a blue asterisk. Furthermore, each sensor with bearing noise $\sigma_{k}=10$ degrees is marked with a green asterisk. In addition, the true target position is depicted as a black circle.

**Figure 5:**
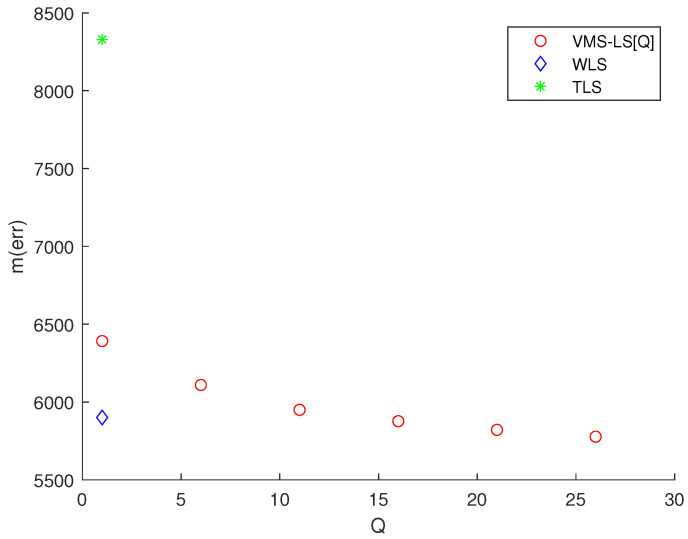
Scenario 2. The variation of M(err) with respect to *Q*. TLS and WLS are plotted at Q=1. Increasing *Q* improves the estimation accuracy of VMS−LS. The algorithm with the lowest M(err) is VMS−LS[26]. This demonstrates the effectiveness of VMS−LS. The estimation error of VMS−LS increased, compared to Figure 2. This shows that as the distance between the sensors and the target increases, the estimation error increases.

Considering Scenario 2, Figure 5 presents the simulation results of various algorithms, mentioned at the beginning of Section 3. This figure shows the variation of M(err) with respect to *Q*. TLS and WLS are plotted at Q=1. Note that using (14), LS solution in (8) is identical to VMS−LS[1]. The estimation error of VMS−LS increased, compared to Figure 2. This shows that as the distance between the sensors and the target increases, the estimation error increases.

**Figure 6:**
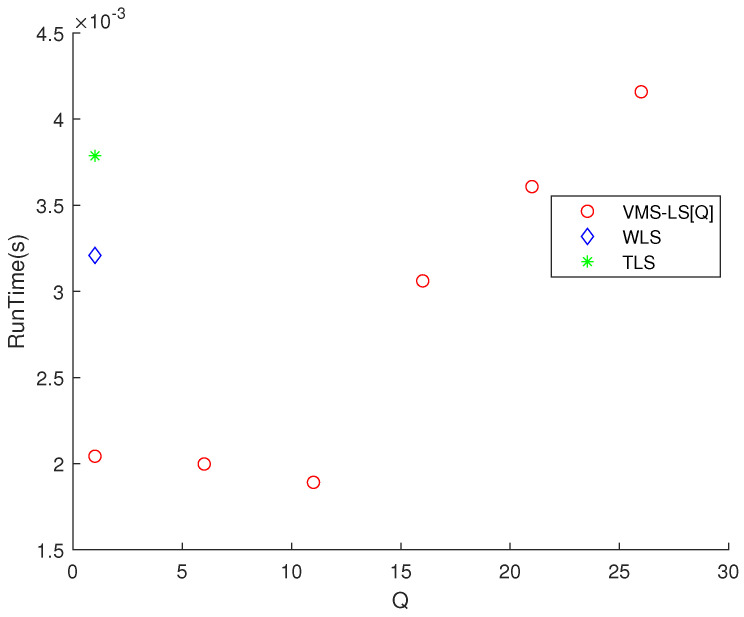
Scenario 2. The variation of RunTime with respect to *Q*. As *Q* increases, computational burden increases in general.

Figure 5 shows that increasing *Q* enhances the estimation accuracy of VMS−LS. The algorithm with the lowest M(err) is VMS−LS[26]. This verifies the performance of VMS−LS proposed in this paper. Figure 6 shows that as *Q* increases, computational burden increases in general.

### 3.3. Scenario 3

**Figure 7:**
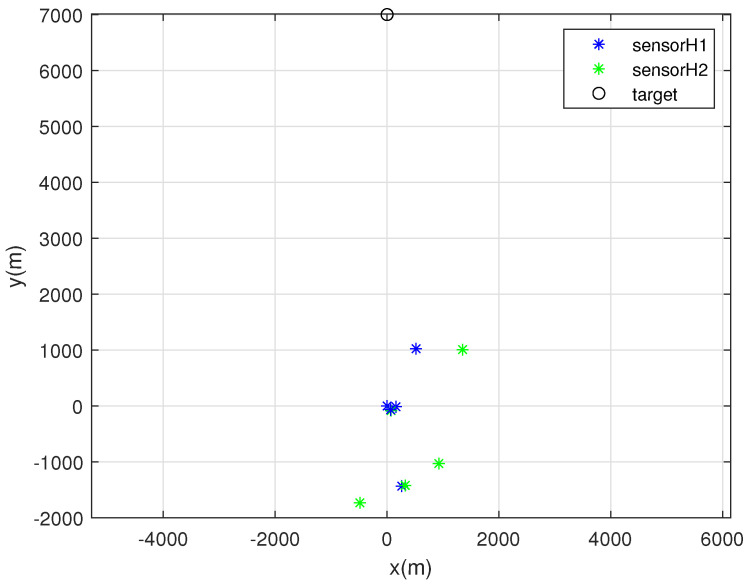
One MC simulation of Scenario 3. At each MC simulation, we randomly deploy 10 sonobuoy sensors inside the circle with radius 2000 m, whose center is at the origin. Each sensor with bearing noise $\sigma_{k}=5$ degrees is marked with a blue asterisk. Furthermore, each sensor with bearing noise $\sigma_{k}=15$ degrees is marked with a green asterisk. In addition, the true target position is depicted as a black circle.

One MC simulation of Scenario 3 is depicted in Figure 7. In this scenario, we deploy many sonobuoy sensors. At each MC simulation, we randomly deploy 10 sonobuoy sensors inside the circle with radius 2000 m, whose center is at the origin. For sensor deployment, we use (27). Each sensor with bearing noise $\sigma_{k}=5$ degrees is marked with a blue asterisk. Furthermore, each sensor with bearing noise $\sigma_{k}=15$ degrees is marked with a green asterisk. Moreover, the true target position is depicted as a black circle.

**Figure 8:**
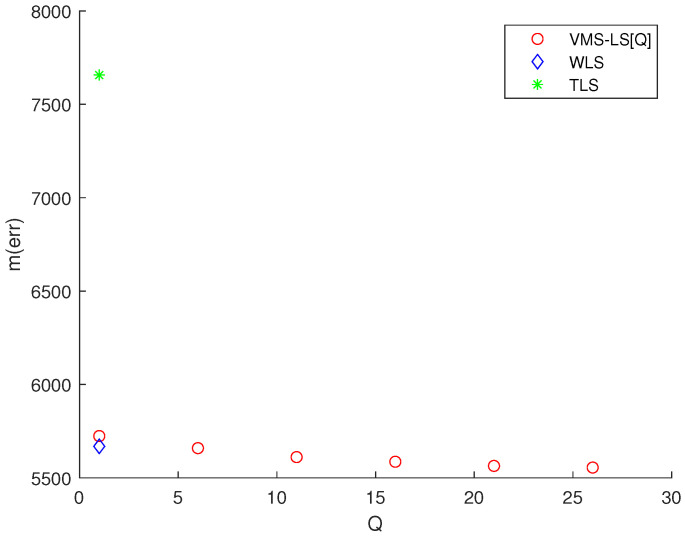
Scenario 3. The variation of M(err) with respect to *Q*. Increasing *Q* improves the estimation accuracy of VMS−LS. The algorithm with the lowest M(err) is VMS−LS[26]. This demonstrates the effectiveness of VMS−LS. The estimation error of VMS−LS decreased, compared to Figure 5. This shows that as the number of sensors increases, the estimation error decreases.

Considering Scenario 3, Figure 8 presents the simulation results of various algorithms, stated at the beginning of Section 3. This figure indicates the variation of M(err) with respect to *Q*. The estimation error of VMS−LS decreased, compared to Figure 5. This shows that as the number of sensors increases, the estimation error decreases.

**Figure 9:**
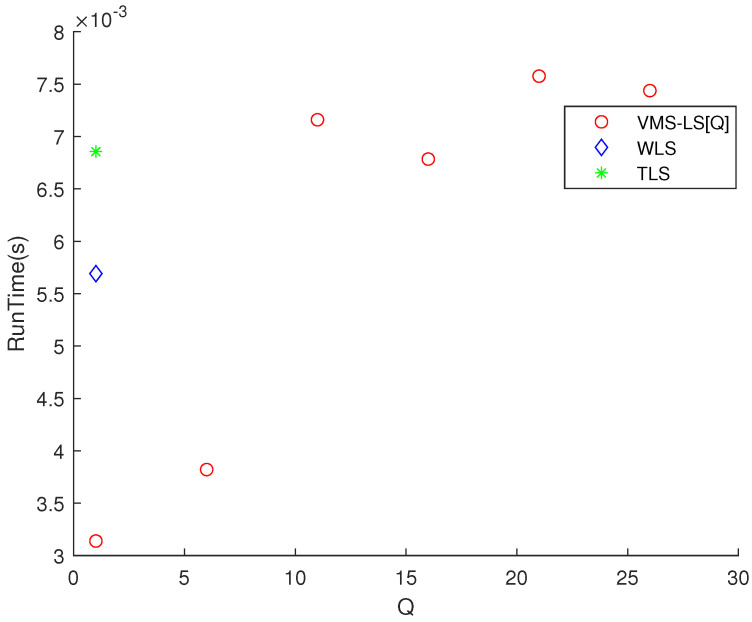
Scenario 3. The variation of RunTime with respect to *Q*. As *Q* increases, computational burden increases in general.

Figure 8 indicates that increasing *Q* improves the estimation accuracy of VMS−LS. The algorithm with the lowest M(err) is VMS−LS[26]. This verifies the performance of using VMS−LS proposed in our paper. Figure 9 shows that as *Q* increases, computational burden increases in general.

### 3.4. Scenario 4

**Figure 10:**
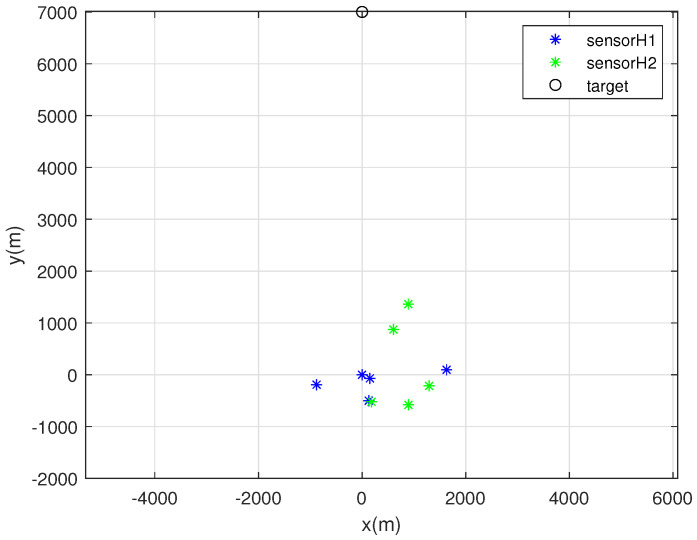
One MC simulation of Scenario 4. At each MC simulation, we randomly deploy 10 sensors inside the circle with radius 2000 m, whose center is at the origin. Each sensor with bearing noise $\sigma_{k}=3$ degrees is marked with a blue asterisk. Each sensor with bearing noise $\sigma_{k}=9$ degrees is marked with a blue asterisk. In addition, the true target position is depicted as a black circle.

One MC simulation of Scenario 4 is depicted in Figure 10. At each MC simulation, we randomly deploy 10 sensors inside the circle with radius 2000 m, whose center is at the origin. For sensor deployment, we use (27). Each sensor with bearing noise $\sigma_{k}=3$ degrees is marked with a blue asterisk. Each sensor with bearing noise $\sigma_{k}=9$ degrees is marked with a blue asterisk. See that we use sensors with small bearing noise, compared to Scenario 3. Moreover, the true target position is depicted as a black circle.

**Figure 11:**
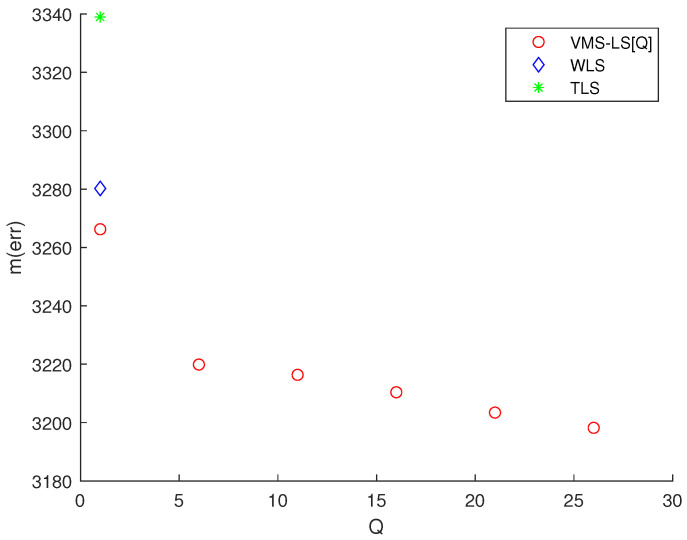
Scenario 4. The variation of M(err) with respect to *Q*. The estimation error of VMS−LS decreased, compared to Figure 8. This shows that as we use sensors with small bearing noise, the estimation error decreases.

Figure 11 presents the simulation results of various algorithms, stated at the beginning of Section 3. This figure indicates the variation of M(err) with respect to *Q*. The estimation error of VMS−LS decreased, compared to Figure 8. This shows that as we use sensors with small bearing noise, the estimation error decreases.

**Figure 12:**
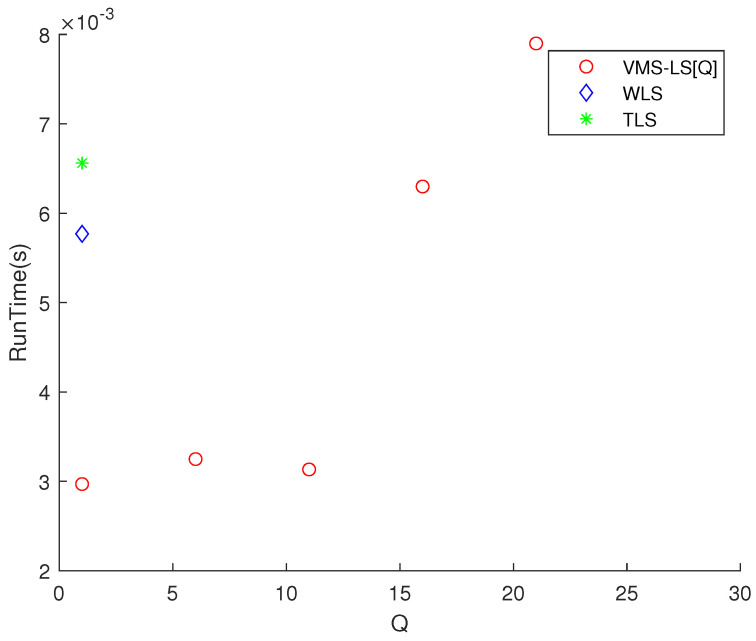
Scenario 4. The variation of RunTime with respect to *Q*. As *Q* increases, computational burden increases in general.

Figure 11 indicates that increasing *Q* improves the estimation accuracy of VMS−LS. The algorithm with the lowest M(err) is VMS−LS[26]. This verifies the performance of using VMS−LS in our paper. Figure 12 shows that as *Q* increases, computational burden increases in general.

## 4. Conclusions

This paper addresses localization of an underwater target based on bearings-only measurements of heterogeneous sonobuoys. Sonobuoys have very low bearing accuracy, such as 10 degrees [8]. We tackle bearings-only localization using heterogeneous sonobuoy sensors, considering the sensing constraints of a sonar sensor. Using MATLAB simulations, we demonstrate the outperformance (considering both location accuracy and time efficiency) of the proposed VMS−LS, by comparing it with other localization methods.

Using MATLAB simulations, VMS−LS[Q] with large *Q* shows the best performance among all algorithms, considering both time efficiency and localization accuracy. Increasing *Q* in VMS−LS enhances the localization accuracy, while increasing the computational load. In the future, we will do experiments using real sonar sensors, in order to verify the performance of the proposed localization more rigorously.

The authors of [36] presented the Gauss Newton (GN) algorithm applied to bearings-only target estimation. The GN method is an iterative method to derive the target estimation. The convergence of the GN estimator is sensitive to the initial guess and the step size [38]. To further decrease the localization error, we can run the GN method which is initialized with the VMS-based solution. In the case where the distance between a sensor and the target estimation from the GN method is bigger than the maximum sensing range, we can get out of the iteration of the GN method. In this way, we avoid a target solution which is too far from a sensor.

This paper addresses a VMS localization method, considers 2D bearings-only localization. In the future, the proposed VMS approach will be applied in various localization schemes, such as 3D angle-of-arrival localization [41], time-of-arrival localization [42,43,44], time-difference-of-arrival localization [45,46,47,48], or doppler-bearings localization [49,50].

## Data Availability

Not applicable.

## References

[1] Han Y., Zheng C., Sun D. Accurate underwater localization using LBL positioning system. Proceedings of the OCEANS 2015—MTS/IEEE Washington.

[2] Magalhães P.E., Cristol X., Ioana C., Fattaccioli D., Mars J. ALMA 2015: Sea Trial of an Underwater Target Localization Technique Using Hausdorff Distance. Proceedings of the 2018 OCEANS—MTS/IEEE Kobe Techno-Oceans (OTO).

[3] Crasta N., Moreno-Salinas D., Bayat B., Pascoal A.M., Aranda J. Range-based underwater target localization using an autonomous surface vehicle: Observability analysis. Proceedings of the 2018 IEEE/ION Position, Location and Navigation Symposium (PLANS).

[4] He C., Wang Y., Yu W., Song L. (2019). Underwater Target Localization and Synchronization for a Distributed SIMO Sonar with an Isogradient SSP and Uncertainties in Receiver Locations. Sensors.

[5] Luo Q., Yan X., Ju C., Chen Y., Luo Z. (2021). An Ultra-Short Baseline Underwater Positioning System with Kalman Filtering. Sensors.

[6] Zhang T., Chen L., Yan Y. (2018). Underwater Positioning Algorithm Based on SINS/LBL Integrated System. IEEE Access.

[7] Maranda B. The statistical accuracy of an arctangent bearing estimator. Proceedings of the Oceans 2003, Celebrating the Past … Teaming toward the Future (IEEE Cat. No. 03CH37492).

[8] Kuzu A., Danış Ü., Kurt E., Karabulut E., Şahinkaya D., Bilgiç E., Kaplaner A., Birecik S., Ozumut B. Laboratory and sea testing of difar sonobuoys. Proceedings of the 2012 IV International Congress on Ultra Modern Telecommunications and Control Systems.

[9] Chang D.H., Park H.B., Na Y.N., Ryu J.H. (2002). Bearing Estimation of Narrow Band Acoustic Signals Using Cardioid Beamforming Algorithm in Shallow Water. J. Acoust. Soc. Korea.

[10] Kuzu A., Daniş Ü., Kurt E., Karabulut E., Şahinkaya D., Bilgiç E., Kaplaner A., Birecik S. Calibration and test of DIFAR sonobuoys. Proceedings of the 2011 IEEE International Symposium on Industrial Electronics.

[11] Ozaki Y., Ozawa J., Taillefer E., Cheng J., Watanabe Y. A simple DoA estimator using adjacent pattern power ratio with switched beam antenna. Proceedings of the 2010 International Conference on Wireless Communications Signal Processing (WCSP).

[12] Kim J. (2021). Direction of Arrival Estimation Using Four Isotropic Receivers. IEEE Instrum. Meas. Mag..

[13] Khan Z.I., Kamal M.M., Hamzah N., Othman K., Khan N.I. Analysis of performance for multiple signal classification (MUSIC) in estimating direction of arrival. Proceedings of the 2008 IEEE International RF and Microwave Conference.

[14] AlShehhi A., Hammadih M.L., Zitouni M., Kindi S.A., Ali N., Weruaga L. (2017). Linear and Circular Microphone Array for Remote Surveillance: Simulated Performance Analysis. arXiv.

[15] Fascista A., Ciccarese G., Coluccia A., Ricci G. (2017). Angle of Arrival- Based Cooperative Positioning for Smart Vehicles. IEEE Trans. Intell. Transp. Syst..

[16] Peach N. (1995). Bearings-only tracking using a set of range-parameterised extended Kalman filters. IEE Proc. Control Theory Appl..

[17] Kim J. (2019). Obstacle Information Aided Target Tracking Algorithms for Angle-Only Tracking of a Highly Maneuverable Target in Three Dimensions. IET Radar Sonar Navig..

[18] Wang X., Chen J.F., Shi Z.G., Chen K.S. (2011). Fuzzy control-based particle filter for maneuvering target tracking. Prog. Electromagn. Res..

[19] Wan M., Li P., Li T. Tracking Maneuvering Target with Angle-Only Measurements Using IMM Algorithm Based on CKF. Proceedings of the 2010 International Conference on Communications and Mobile Computing.

[20] Ristic B., Arulampalam M.S. (2003). Tracking a manoeuvring target using angle-only measurements: Algorithms and performance. Signal Process..

[21] Li L., Xie W., Liu Z. (2015). Bearings-only maneuvering target tracking based on truncated quadrature Kalman filtering. AEU—Int. J. Electron. Commun..

[22] Pignol A., Jauffret C., Pillon D. A statistical fusion for a leg-by-leg bearings-only TMA without observer maneuver. Proceedings of the 2010 13th International Conference on Information Fusion.

[23] Kim J., Suh T., Ryu J. (2017). Bearings-only target motion analysis of a highly manoeuvring target. IET Radar Sonar Navig..

[24] Quan H., Li J., Zhang X. Maneuvering target tracking with ESM sensor. Proceedings of the International Conference on on Soft Computing in Information Communication Technology (SCICT 2014).

[25] Kim J. (2019). Observer manoeuvre control to track multiple targets considering Doppler-bearing measurements in threat environments. IET Radar Sonar Navig..

[26] Nardone S., Lindgren A., Gong K. (1984). Fundamental properties and performance of conventional bearings-only target motion analysis. IEEE Trans. Autom. Control.

[27] Karlsson R., Gustafsson F. (2005). Recursive Bayesian estimation: Bearing-only applications. IEE Proc. Radar Sonar Navig..

[28] Nardone S.C., Aidala V.J. (1981). Observability Criteria for Bearings-Only Target Motion Analysis. IEEE Trans. Aerosp. Electron. Syst..

[29] Yang H., Wang Y. (2019). Formation Optimization and Control for Maneuvering Target Tracking by Mobile Sensing Agents. IEEE Access.

[30] Lingren A.G., Gong K.F. (1978). Position and Velocity Estimation via Bearing Observations. IEEE Trans. Aerosp. Electron. Syst..

[31] Markovsky I., Van Huffel S. (2007). Overview of total least-squares methods. Signal Process..

[32] Gavish M., Weiss A. (1992). Performance analysis of bearing-only target location algorithms. IEEE Trans. Aerosp. Electron. Syst..

[33] Dogancay K. (2006). Bias compensation for the bearings-only pseudolinear target track estimator. IEEE Trans. Signal Process..

[34] Kay S. (1993). Fundamentals of Statistical Signal Processing, Volume I: Estimation Theory.

[35] Doundefinedançay K. (2005). Bearings-Only Target Localization Using Total Least Squares. Signal Process..

[36] Torrieri D.J. (1984). Statistical Theory of Passive Location Systems. IEEE Trans. Aerosp. Electron. Syst..

[37] Luo J.A., Shao X.H., Peng D.L., Zhang X.P. (2019). A Novel Subspace Approach for Bearing-Only Target Localization. IEEE Sens. J..

[38] Le Cadre J., Jaetffret C. (1999). On the convergence of iterative methods for bearings-only tracking. IEEE Trans. Aerosp. Electron. Syst..

[39] Li W., Tang Q., Huang C. (2017). A New Close Form Location Algorithm with AOA and TDOA for Mobile User. Wirel. Pers. Commun..

[40] Ho K.C., Chan Y.T. (2006). An asymptotically unbiased estimator for bearings-only and doppler-bearing target motion analysis. IEEE Trans. Signal Process..

[41] Xu S., Dogancay K. (2017). Optimal Sensor Placement for 3-D Angle-of-Arrival Target Localization. IEEE Trans. Aerosp. Electron. Syst..

[42] Chan Y.T., Tsui W.Y., So H.C., Ching P.-C. (2006). Time-of-arrival based localization under NLOS conditions. IEEE Trans. Veh. Technol..

[43] Lay K.T., Chao W.K. Mobile Positioning Based on TOA/TSOA/TDOA Measurements with NLOS Error Reduction. Proceedings of the 2005 International Symposium on Intelligent Signal Processing and Communication Systems.

[44] Guvenc I., Chong C.C. (2009). A Survey on TOA Based Wireless Localization and NLOS Mitigation Techniques. IEEE Commun. Surv. Tutor..

[45] Zhang L., Zhang T., Shin H.S., Xu X. (2021). Efficient Underwater Acoustical Localization Method Based on Time Difference and Bearing Measurements. IEEE Trans. Instrum. Meas..

[46] Musicki D., Kaune R., Koch W. (2009). Mobile Emitter Geolocation and Tracking Using TDOA and FDOA Measurements. IEEE Trans. Signal Process..

[47] Boccadoro M., Angelis G.D., Valigi P. (2012). TDOA positioning in NLOS scenarios by particle filtering. Wirel. Netw..

[48] Montminy M.B. (2012). Passive Geolocation of Low-Power Emitters in Urban Environments Using TDOA.

[49] Kim J. (2020). 3D path planner of an autonomous underwater vehicle to track an emitter using frequency and azimuth–elevation angle measurements. IET Radar Sonar Navig..

[50] Li X., Zhao C., Yu J., Wei W. (2019). Underwater Bearing-Only and Bearing-Doppler Target Tracking Based on Square Root Unscented Kalman Filter. Entropy.

